# *Treponema pallidum* infection predicts sexually transmitted viral infections (hepatitis B virus, herpes simplex virus-2, and human immunodeficiency virus) among pregnant women from rural areas of Mwanza region, Tanzania

**DOI:** 10.1186/s12884-019-2567-1

**Published:** 2019-10-29

**Authors:** Gilbert Ng’wamkai, Kalista V. Msigwa, Damas Chengula, Frank Mgaya, Clotilda Chuma, Betrand Msemwa, Vitus Silago, Mtebe Majigo, Stephen E. Mshana, Mariam M. Mirambo

**Affiliations:** 1 Department of Obstetrics and Gynecology, Bugando Medical Centre, P.O. Box 370, Mwanza, Tanzania; 2 Department of Microbiology and Immunology, Weill Bugando School of Medicine, Catholic University of Health and Allied sciences, P.O. Box 1464, Mwanza, Tanzania; 3 Institute of Allied Health Sciences, Catholic University of Health and Allied sciences, P.O. Box 1464, Mwanza, Tanzania; 4 Department of Microbiology and Immunology, Muhimbili University of Health and Allied Sciences, P.O. Box 65001, Dar es Salaam, Tanzania

**Keywords:** Herpes simplex-2, Hepatitis B, Hepatitis C, HIV, Mwanza, Rural

## Abstract

**Background:**

Sexually transmitted infections (STIs) is a global health problem with increased risk and morbidities during pregnancy. This study investigated the magnitude of viral STIs among pregnant women from three rural hospitals/clinics providing antenatal care in Mwanza region, Tanzania.

**Methods:**

Between February and May 2018, a total of 499 pregnant women were enrolled and tested for Human immunodeficiency virus (HIV), Herpes simplex virus-2 (HSV-2), Hepatitis B virus (HBV) and Hepatitis C virus (HCV) using rapid immunochromatographic tests and for syphilis using non-treponemal and treponemal antibody test.

**Results:**

The median age of enrolled women was 25 (IQR: 22–31) years. Seventy eight (15.6, 95% CI: 12–18) of women tested had at least one sexually transmitted viral infection. Specific prevalence of HIV, HBV, HCV, HSV-2 IgG and HSV-2 IgM were found to be 25(5.0%), 29(5.8%), 2(0.4%), 188(37.7%) and 24(4.8%), respectively. The odds of having viral infection was significantly high among women with positive *T. pallidum* serostatus (adjusted odd ratio (aOR): 3.24, 95%CI; 1.2–85). By multivariable logistic regression analysis, history of STIs predicted HSV-2 IgM seropositivity (aOR: 3.70, 95%CI: 1.43–9.62) while parity (aOR: 1.23, 95%CI: 1.04–1.46) predicted HBV infection and syphilis positive results (aOR: 8.63, 95%CI: 2.81–26.45) predicted HIV infection.

**Conclusion:**

A significant proportion of pregnant women in rural areas of Mwanza region has at least one sexually transmitted viral infection which is independently predicted by positive *T. pallidum* serostatus. The strengthening and expansion of ANC screening package to include screening of STIs will ultimately reduce the viral STIs among pregnant women hence reduce associated morbidities and mortalities.

## Background

Sexually transmitted infections (STI) particularly those caused by viruses have been associated with a number of adverse pregnancy outcomes including stillbirth, spontaneous abortion, preterm births, prematurity, postpartum endometritis and low birth weight among other various long term sequelae in surviving neonates [1, 2]. Herpes simplex virus type 2 (HSV-2) is the main causative agent of genital ulcer diseases worldwide [3]. It can be transmitted vertically from mother to the fetus and it is highly prevalent in the sub-Saharan Africa whereby it has been found to cause severe illness to the neonates [4–7].

On the other side, a previous study reported that nearly 90% of pregnant women living with HIV in the world are from sub-Saharan Africa [8, 9]. Challenges associated with effective prevention of mother-to-child transmission of HIV (PMTCT) include adherence and failure in identifying HIV-infected mothers [8], making HIV still a problem in these countries. Regarding viral hepatitis, Hepatitis B virus (HBV) and hepatitis C viruses (HCV) are major public health concern worldwide with high prevalence in the sub-Saharan Africa and Asia [10, 11]. It is estimated that, over 1 million deaths related to chronic liver disease are due to HBV [12]. Most of individuals in endemic areas are infected vertically or during childhood [10]. Similarly, HCV infection are common in many countries worldwide [11]. The World Health Organization (WHO) estimates that 3% of the world’s populations are chronically infected with HCV with high prevalence reported in the sub-Saharan Africa [13, 14].

The current practice regarding antenatal screening in Tanzania includes routine screening of HIV and *T. pallidum* infections while the care for other STIs like HSV-2, HBV and HCV mostly rely on symptoms. Nevertheless, in many developing countries particularly in rural settings, it is uncommon to visit health facilities unless having signs and symptoms of disease and most of these STIs are asymptomatic [15]. Due to the current situation regarding antenatal screening of these infections in Tanzania, data regarding the magnitude of these infections among pregnant women in rural setting are scarce. Lack of these data hinders introduction of the appropriate strategies to prevent these infections especially in rural areas. This study was conducted to establish the magnitude of sexually transmitted viral infections among pregnant women in Tanzania. The data might help to come up with recommendations on the current screening protocol and control strategies.

## Methods

### Study design and duration

A cross sectional hospital based study involving 499 pregnant women attending three rural antenatal clinics (Magu, Karume and Sengerema) in Mwanza region was conducted between February and May 2018.

### Study area

The study was conducted in Sengerema, Ilemela and Magu districts in Mwanza region, Tanzania. (http://www.jgid.org/viewimage.asp?img=JGlobalInfectDis_2010_2_3_216_68530_u3.jpg). Mwanza is located in the North-Western zone of Tanzania, with a population of 2,772,509 [16]. Sengerema Designated Ditrict hospital (DDH) has a capacity of 320 beds and attends approximately 30–40 pregnant women daily at its antenatal clinic [17], it is located 60 km from Mwanza city. Magu District Hospital (MDH) is 60 km from Mwanza city and has capacity of 200 beds and attends approximately 30–40 pregnant women daily in the antenatal clinic. Karume Health Centre (KHC) is located in Ilemela district 20 km from Mwanza city and serves about 20–30 pregnant women daily. The antenal atendance in Mwanza region in 2018 is about 73% with 83% delivery in health facilities (www.dhis.moh.go.tz).

### Study population and selection criteria

All pregnant women at different ages and gestation ages attending at KHC, Sengerema DDH and MDH antenatal clinics were included in the study after obtaining informed consent.

### Sample size estimation and sampling technique

The sample was calculated by Kish Leslie formula for observational study [18] using the prevalence of 50% to get the minimum sample size of 384 participants, however, 499 pregnant women were enrolled. Purposive sampling was used to select the study areas and the study participants were enrolled serially as they come to a clinic until the desired sample size of that clinic was reached. The sample size from each clinic was calculated based on the proportion of women attending specific clinic daily.

### Data and samples collection

A pre-tested structured data collection tool which comprised both open and close ended questions was used to collect required data (Additional file 1). Data collected included demographic and clinical data such signs and symptoms of STIs. Individual face to face interview was used to obtain these data from a study participant. Antenatal cards were reviewed to get information such as antenatal visits profile, gestation age and important antenatal investigations such as syphilis and HIV testing.

The first trimester was defined as ≤13 weeks, second trimester 13 - ≤ 26 weeks and third trimester more than 26 weeks of gestation. Data were collected by nurses in case the patient required special attention was reviewed by doctors present in the respective hospital or the OB/GY resident. About 4-5mls of venous blood sample was collected from each of enrolled participant and placed in plain vacutainer tubes (Becton, Dickinson and Company, USA). Samples were transported to the Catholic University of Health and allied sciences for processing. Sera were extracted and stored in cryovials at -40C until analysis.

### Laboratory procedures

Samples were tested as per national HIV diagnosis algorithm using SD Bioline and Unigold as previously, described [19] to establish HIV serostatus. Detection of HSV-2 virus specific IgM and IgG antibodies was done by using rapid immunochromatographic test as per manufacturer’s instructions (Exact Diagnostic Devices -USA). The test has a sensitivity of 95% and specificity of 94.7%. Hepatitis B surface antigen HBsAg (Accu-Tell, Beijing, China) and HCV antibodies were tested using rapid immunochromatography tests as per manufacturer’s instructions (ACON laboratories, Inc., CA92121, USA). Syphilis was detected using non-treponemal test (Standard diagnostics-Inc, Korea) and all positive samples were confirmed using *Treponema pallidum* Hemagglutination test (Chronolab systems, Barcelona, Spain).

### Data analysis

Data collected were entered into a computer using Microsoft Office Excel 2007 and analyzed by using STATA version 13 (STATA Corp LP, USA). In the present study any patient with history of vaginal discharge or genital ulcer or clinically treated as case of STIs in the past was considered to have history of STIs. Presence of at least one viral infections was defined as being positive to HSV2 IgM antibodies or positive HIV status or positive HBsAg or positive HCV antibodies. Categorical variables (marital status, education level, occupation, history of STIs, use of condoms etc.) were summarized as proportions and were analyzed using the Pearson’s Chi-square test to observe the significant differences in distribution of viral infections among various groups like age, education levels etc. Continuous variables (age, gestation age, parity, number of sexual partners etc.) were summarized as median with inter-quartile range. Univariable and multivariable logistic regression model was used to determine the factors associated with HSV-2, HIV and HBV positivity. Variables with *p*-value less than 0.2 on univariable regression model were subjected into the backward multivariable logistic regression analysis adjusted by age. Odds ratio and 95% confidence interval were noted. Variables with p-value of less than 0.05 were considered statistically significant.

## Results

### Sociodemographic, relationship and sexual history characteristics of 499 pregnant women attending ANCs in Mwanza – rural Tanzania

A total of 499 pregnant women with the median age of 25 (IQR: 22–31) years were enrolled. Of these women, 98, 200 and 201 were from Karume clinic (Ilemela), Magu district hospital and Sengerema district hospital, respectively. Most of the participants were in the second 257(49.5%) and third 222(46.5%) trimesters with the median gestation age of 25(IQR: 20–32) weeks. Approximately two-thirds (63.9%) had completed primary school while (17.8%) of the women had never attended any formal school. The majority of women 464 (93%) were married and more than half 277 (55.6%) were peasants. Approximately more than half 266 (53.3%) reported having more than one sexual partner in lifetime and less than one-fourth of women 109 (21.8%) knew that their partner had other partner(s) outside of their relationship**.** Of the 499 participants, 20 (4%) were *T. pallidum* (syphilis) seropositive (Table 1).

**Table 1:** Sociodemographic, relationship, sexual characteristics and clinical signs among 499 pregnant women attending ANCs in Mwanza-rural Tanzania

Characteristics	Frequency/Median (IQR)	Percentages (%)
Age (years)	25 [IQR: 22–25]	
Gestation age (weeks)	25[IQR:20–32]	
Age of 1st sexual intercourse	17[IQR16–18]	
Parity	1[IQR 0–3]	
Number of sex partners	2[IQR1–4]	
Condom use		
Yes	111	22.2
No	388	77.8
Education level		
Never attended	89	17.84
Primary	319	63.93
Secondary	91	18.24
Occupation		
Peasant	227	55.51
House wife	79	12.83
Business/employed	143	28.66
Parity		
Primigravida	130	26.05
Multipara	262	52.51
Grandmultipara	107	21.44
Gestation age		
First trimmest	20	4.00
Second trimester	257	51.50
Third trimester	222	44.49
Marital status		
Married	464	92.99
Not married	35	7.01
Partner has other women outside relationship		
Yes	109	21.84
No	390	78.16
More than two Sexual partners		
Yes	266	53.31
No	233	46.69
Syphilis status		
Negative	479	95.99.
Positive	20	4.01
H/Vagina discharge		
Yes	92	18.44
No	407	81.56
H/Dysuria		
Yes	174	34.87
No	325	65.13
H/Dyspareunia		
Yes	109	21.84
No	390	78.16
H/Lower abdominal Pain		
Yes	249	49.90
No	250	50.10
H/Genital ulcer		
Yes	28	5.61
No	471	94.39

### History of STIs symptoms and risk behaviour among 499 pregnant women attending ANCs in rural areas of Mwanza region Tanzania

Among 499 pregnant women enrolled, 92 (18.4%) were found to have history of STIs and almost half of the women 249 (49.9%) reported a history of lower abdominal pain. A total of 174 (34.9%), 109 (21.8%) and 28 (5.6%) women reported history of dysuria, dyspareunia and genital ulcers, respectively. In addition, more than three quarters 388(77.8%) of women reported no history of condom use during sex (Table 1).

### Prevalence of HSV-2, HIV, HBV and HCV among 499 pregnant women attending ANCs in Mwanza- rural Tanzania

Seventy eight (15.6, 95% CI: 12–18) of women tested, had at least one sexually transmitted viral infection. A total of 188(37.7, 95% CI: 33–41) were found to be HSV-2 IgG seropositive while 24 (4.8 95% CI: 2–6) wereHSV-2 IgM seropositive. On Wilcoxon Ranksum (Man Whitney) test there was no significant difference in age between those who were HSV-2 IgG seropositive and HSV-2 IgG seronegative (26, IQR: 21–31 vs. 25, IQR: 22–31, *p* = 0.455). However, the HSV-2 IgG seroprevalence was significantly high among women aged below 20 years compared to women aged above 20 years [45 (47.4%) vs. 143(35.4), *p* = 0.030]. In addition, 8 women who were HSV-2 IgG seropositive were found to have genital ulcer whereby 4 of them were *T. pallidum* (syphilis) seropositive. Regarding HSV-2 IgM seropositivity, there was no significant difference in age between IgM seropositive women and seronegative women (25, IQR: 22–28 vs. 26, IQR: 21–31, *p* = 0.699).

**Fig. 1:**
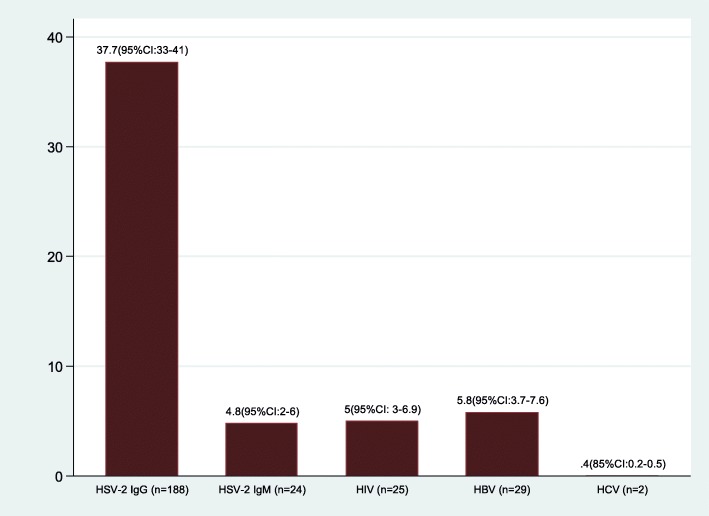
Prevalence of sexually transmitted viral infections among 499(N) pregnant women in rural areas of Mwanza region. Letter “n” refers to positive samples for each virus

The prevalence of HBV was found to be 29(5.8, 95% CI: 3.7–7.6). The median age of HBV positive women was 30 (IQR: 21–33) years compared to HBV negative women 25 (IQR: 22–31), *p* = 0.082. The seroprevalence of HIV was 25(5.0, 95% CI: 3.0–6.9). The median age of HIV seropositive women was significantly high compared to their counterparts [28 (IQR: 25–32) vs. 25 (IQR: 21–31), *p* = 0.032]. Overall, seroprevalence of HCV were found to be 2(0.4, 95% CI: 0.2–0.5) (Fig. 1). These two patients were HIV, HBV, HSV IgM and syphilis negative but were positive for HSV IgG antibodies.

### Factors associated with HSV-2 IgG and HSV-2 IgM seropositivity among 499 pregnant women attending ANCs in Mwanza- rural Tanzania

Regarding HSV-2 IgG seropositivity, on univariable analysis, only age at first sex (aOR: 0.917, 95%CI: 0.84–0.99, *p* = 0.028) was found to be associated with IgG seropositivity. However, the history of STIs (aOR: 1.45, 95%CI: 0.99–2.12, *p* = 0.053) had borderline significance on multivariable analysis (Table 2). In addition, on univariable analysis, history of STIs (OR: 3.47, 95% CI: 1.36–8.91, *p* = 0.009) was significantly associated with specific HSV-2 IgM seropositivity and remained significant on multivariable logistic regression analysis (aOR: 3.70, 95% CI: 1.43–9.62, *p* = 0.007) (Table 3).

**Table 2:** Univariable and multivariable logistic regression analysis of the factors associated with specific HSV-2 IgG seropositivity among 499 pregnant women attending ANC’s in Mwanza-rural Tanzania

Characteristics	HSV IgG Seropositivity (%)	Univariable	*P* – value	Multivariable	*P* – value
Age	26 [IQR 21–31]	0.99[0.97–1.02]	0.678	1.00[0.97–1.03]	0.895
Parity	1.5 [IQR 0–3]	0.98 [1.089–1.076]	0.676		
Gestational age	24 [IQR 20–32]	0.99 [0.97–1.02]	0.562		
Number sexual of partners	2 [IQR 1–4]	1.03 [0.98–1.08]	0.315		
*Age at first sex	17[IQR:15–18]	0.917[0.84–0.99]	0.028	0.92[0.85–1.00]	0.056
*Occupation					
Employed (143)	46(32.17)	ref			
Peasant (277)	109(39.35)	1.37[0.89–2.09]	0.149	1.42[0.92–2.18]	0.113
House wife (79)	33(41.77)	1.52[0.86–2.67]	0.153	1.44[0.80–2.59]	0.221
Education					
Secondary (91)	28 (30.77)	ref			
Primary (319)	130(40.75)	1.55[0.94–255]	0.086		
Never attended (89)	30(33.71)	1.44[0.61–2.14]	0.673		
Marital status					
Married (464)	171(36.85)	ref			
Not married (35)	17(48.57)	1.62[0.81–3.22]	0.171		
*History of genital ulcer					
No (452)	175(38.72)	ref			
Yes (47)	13(27.66)	0.61[0.31–1.18]	0.140	0.53[0.26–1.06]	0.074
Genital ulcers					
Absent (471)	180(38.22)	ref			
Present (28)	8 (28.57)	0.65 [0.28–1.49]	0.309		
*History of STIs					
No (261)	90(34.48)	ref			
Yes (238)	98 (41.18)	1.33 [0.93–1.91]	0.124	1.45[0.88–2.12]	0.053
*HIV status					
Negative (474)	182(38.40)	ref			
Positive (25)	6 (24%)	0.51[0.19–1.29]	0.155	0.55[0.21–1.42]	0.218
Syphilis status					
Negative (479)	178(37.16)	ref			
Positive (20)	10(50%)	1.69[0.69–4.14)	0.250		

*Age of first sex, occupation, history of genital ulcers, history of STIs and HIV status were subjected on multivariable analysis adjusted by age, ref = reference

**Table 3:** Univariable and multivariable logistic regression analysis of the factors associated with specific HSV-2 IgM seropositivity among 499 pregnant women attending ANC’s in Mwanza rural Tanzania

Characteristics	HSV IgM Seropositivity %	Univariable OR (95%CI)	*P* value	Multivariable OR (95%CI)	*P* value
Age	25[IQR:22–28]	0.98[0.92–1.05]	0.595	0.99[0.93–1.06]	0.884
Parity	1[IQR:0–3]	0.93[0.75–1.16]	0.515		
Gestation age	25.5[IQR:20–30]	0.10[0.94–1.05]	0.906		
Number of sexual partner	3[IQR:1–5]	0.10[0.89–1.11]	0.993		
Age at first sex	17[IQR:15–18]	0.94[0.72–1.12]	0.509		
*Occupation					
Employed (143)	5(3.50%)	ref			
Peasant (277)	13(4.69%)	1.35[0.47–3.90]	0.567	1.41[0.48–4.14]	0.525
House wife (79)	6(7.59%)	2.27[0.67–7.69]	0.188	2.29[0.66–7.10]	0.192
Education					
Secondary (91)	2(2.2%)	ref			
Primary (319)	15(4.7%)	2.20[0.49–9.78]	0.302		
Never attended (89)	7(7.87%)	3.80[0.77–18.81]	0.102		
*Marital status					
Married (464)	21(4.53%)	ref			
Not married (35)	3(8.5%)	1.98[0.56–6.98]	0.289	2.12[0.57–7.83]	0.260
History of genital ulcer					
No (452)	23(5.1)	ref			
Yes (47)	1(2.1)	0.41[0.1–3.07]	0.312		
Genital ulcers					
Absent (471)	24(5.1)	ref			
Present (28)	0 (0.0)	omitted	omitted		
*History of STIs					
No (261)	6(2.30)	ref			
Yes (238)	18(7.56%)	3.47[1.36–8.91]	0.009	3.7[1.43–9.62]	0.007
HIV status					
Negative (474)	23(4.8)	ref			
Positive (25)	1 (4.0)	0.817[0.10–6.30]	0.846		
Syphilis status					
Negative (479)	23(4.8%)	ref			
Positive (20)	1(5.0%)	1.04(0.13–8.13)	0.968		

*Occupation, marital status and history of STIs were subjected on multivariable analysis adjusted by age

### Factors associated with HBV and HIV among 499 pregnant women

Increase in parity (aOR: 1.23, 95% CI: 1.04–1.46, *p* = 0.015) significantly predicted HBsAg positivity on multivariable logistic regression analysis (Table 4). Regarding HIV seropositivity, on univariable analysis, parity (OR: 1.2, 95% CI: 1.08–1.51, *p* = 0.03), age (aOR: 1.06, 95% CI: 1.00–1.13, *p* = 0.044), and positive syphilis serostatus (OR: 10.37, 95% CI: 3.59–29.97, *p* < 0.001) were found to be significantly associated with HIV seropositivity. Only positive syphilis serostatus (aOR: 8.63, 95% CI: 2.81–26.45, *p* < 0.001) predicted HIV seropositivity (Table 5).

**Table 4:** Univariable and multivariable logistic regression analysis of the factors associated with specific HBsAg positivity among 499 pregnant women attending ANC’s in Mwanza-rural Tanzania

Characteristics	HBsAg IgG Seropositivity (%)	Univariable	*P* – value	Multivariable	*P* – value
*Age	21 [IQR 21–33]	1.06[1.00–1.12]	0.034		
Parity	3 [IQR 1–5]	1.2 [1.08–1.51]	0.003	1.23[1.04–1.46]	0.015
Gestational age	22 [IQR 20–32]	0.99 [0.94–1.03]	0.679		
Number sexual of partners	3 [IQR 1–5]	1.04 [0.96–1.12]	0.304		
Age at first sex	17[IQR:15–20]	1.02[0.97–1.06]	0.319		
Occupation					
Employed (143)	7(4.90)	ref			
Peasant (277)	21(7.58)	1.59[0.66–3.84]	0.299	1.4[0.57–3.42]	0.460
House wife (79)	1(1.27)	0.24[0.03–2.06]	0.197	0.30[0.03–2.52]	0.269
Education					
Secondary (91)	4 (4.40)	ref			
Primary (319)	19(5.96)	1.37[0.46–4.15]	0.570		
Never attended (89)	6(6.74)	1.57[0.42–5.77]	0.495		
Marital status					
Married (464)	28(6.03)	ref			
Not married (35)	1(2.86)	2.18[0.29–16.54]	0.450		
History of genital ulcer					
No (452)	27(5.97)	ref			
Yes (47)	2(4.26)	0.70[0.16–3.03]	0.634		
Genital ulcers					
Absent (471)	28(5.94)	ref			
Present (28)	1 (3.57)	0.58 [0.08–4.47]	0.606		
History of STIs					
No (261)	13(4.98)	ref			
Yes (238)	16 (6.72)	1.37 [0.65–2.92]	0.408		
HIV status					
Negative (474)	29(6.12)	ref			
Positive (25)	0 (0.00)	Omitted			
Syphilis status					
Negative (479)	28(5.85)	ref			
Positive (20)	1(5)	0.85[0.11–6.56)	0.874		

*Age was not subjected in the multivariable analysis due to its collinearity with parity

**Table 5:** Univariable and multivariable logistic regression analysis of the factors associated with specific HIV seropositivity among 499 pregnant women attending ANC’s in Mwanza-rural Tanzania

Characteristics	HIV Seropositivity (%)	Univariable	*P* – value	Multivariable	*P* – value
Age	28 [IQR 25–32]	1.06[1.00–1.13]	0.044	1.02[0.93–1.13]	0.571
*Parity	3 [IQR 1–5]	1.2 [1.08–1.51]	0.003	1.09[0.80–1.47]	0.559
Gestational age	22 [IQR 20–32]	0.99 [0.94–1.03]	0.679		
Number sexual of partners	3 [IQR 1–5]	1.04 [0.96–1.12]	0.304		
Age at first sex	17[IQR:15–20]	1.02[0.97–1.06]	0.319		
Occupation					
Employed (143)	7(4.9)	ref			
Peasant (277)	16(5.8)	1.19[0.47–2.94]	0.707		
House wife (79)	2(2.5)	0.50[0.10–2.48]	0.401		
Education					
Secondary (91)	3 (3.3)	ref			
Primary (319)	15(4.7)	1.45[0.40–5.11]	0.566		
Never attended (89)	7(7.9)	2.50[0.62–10.00]	0.194		
Marital status					
Married (464)	28(6.03)	ref			
Not married (35)	1(2.86)	2.18[0.29–16.54]	0.450		
History of genital ulcer					
No (452)	22(4.9)	ref			
Yes (47)	3(6.4)	1.33[0.38–4.63]	0.651		
Genital ulcers					
Absent (471)	22(4.7)	ref			
Present (28)	3 (10.7)	2.44 [0.68–8.73]	0.167	1.65[0.40–6.80]	0.485
History of STIs					
No (261)	13(4.98)	ref			
Yes (238)	16 (6.72)	1.37 [0.65–2.92]	0.408		
*Syphilis status					
Negative (479)	19(4.0)	ref			
Positive (20)	6 (30)	10.37[3.59–29.97]	< 0.001	8.63[2.81–26.45]	< 0.001

*Parity and syphilis status were subjected on multivariable analysis adjusted by age

### Factors associated with at least one viral infections among 499 pregnant women

The prevalence of viral STIs was significantly higher in *T. pallidum* seropositive women than seronegative women (40% vs. 14.6%, *p* = 0.004). Age, parity, education, marital status, syphilis serostatus were found to be significantly associated with at least one viral STIs (Table 6). Only positive syphilis serostatus (aOR: 3.25, 95% CI: 1.23–8.57, *p* = 0.016) independently predicted at least one viral infection (Table 6).

**Table 6:** Univariable and multivariable logistic regression analysis of the factors associated with at least one viral infection among 499 pregnant women attending ANC’s in Mwanza rural Tanzania

Characteristics	Viral infection	Univariable OR (95%CI)	*P* value	Multivariable OR (95%CI)	*P* value
Age	27[IQR:22–32]	1.04[1.0–1.08]	0.042	1.00[0.94–1.06]	0.918
Parity	2[IQR: 1–4]	1.15[1.02–1.29]	0.016		
Gestation age	22[IQR:20–30]	0.97[0.94–1.00]	0.085		
Number of sexual partner	2.5[IQR:1–5]	1.03[0.97–1.09]	0.249		
Age at first sex	17[IQR:16–19]	1.01[0.92–1.11]	0.790		
Occupation					
Employed (143)	19(13.29%)	ref			
Peasant (277)	50(18.05%)	1.44[0.81–2.55]	0.213		
House wife (79)	9(11.90%)	0.84[0.36–1.95]	0.684		
*Education					
Secondary (91	9(9.89%)	ref			
Primary (319)	50(15.7%)	1.69[0.79–3.59]	0.169	1.28[0.57–2.83]	0.539
Never attended (89)	19(21.3%)	2.470[0.38–18.1.05]	0.038	1.58[0.63–4.00]	0.330
Marital status					
Married (464)	27(20.0%)	ref			
Not married (35)	71(15.30%)	1.38[0.58–3.29]	0.046		
History of genital ulcer					
No (471)	71(15.7%)	ref			
Yes (28)	7(14.9%)	0.93[0.40–2.17]	0.884		
Genital ulcers					
Absent (471)	74(15.7)	ref			
Present (28)	4 (14.3)	0.89 [0.30–2.65]	0.840		
^a^History of STIs					
No (261)	35(13.4)	ref			
Yes (238)	43(18.1)	1.42[0.87–2.31]	0.154	Omitted	
*Syphilis status					
Negative (479)	70(14.6%)	ref			
Positive (20)	8(40.0%)	3.89(1.53–9.87)	0.004	3.25[1.23–8.57]	0.016

^a^Omitted because of collinearity. Syphilis status, education and history of STIs were subjected on multivariable analysis adjusted by age

## Discussion

Sexually transmitted infections are common cause of morbidities and adverse pregnancy outcomes among pregnant women worldwide with the high prevalence reported in low-income countries (LICs) [20]. This study assessed the prevalence of sexually transmitted viral infections (HIV, HSV-2, HBV and HCV) among pregnant women in rural areas of Mwanza region. In this study it was observed that a significant proportion of pregnant women in rural areas of Mwanza region had at least one sexually transmitted viral infections.

### Prevalence and factors associated with HSV-2 seropositivity among pregnant women in rural areas of Mwanza region

Overall seroprevalence of specific HSV-2 IgG and IgM antibodies among pregnant women in this study was high, which is similar to a previous study among adolescent pregnant women in the same settings [21]. Furthermore, this observation is similar to another study which was done in Ethiopia in the same population [22]. Moreover, in the current study, there was significant difference in HSV-2 IgG seroprevalence among women aged below 20 years compared to those aged above 20 years. When compared with a previous study by Hokororo et al, the IgG seroprevalence among women aged below 20 years in the current study was significantly high (*p* = 0.019) [21]. These findings are contrary to previous studies which showed that seroprevalence of HSV-2 tend to increase with age [23, 24]. The possible explanation could be sensitivity and specificity of the test used in these studies whereby, in the current study rapid immunochromatographic test was used with sensitivity and specificity of 95 and 94.7%, respectively. When compared to a previous study in Zimbabwe, the reported seroprevalence in the current study is slightly low; nevertheless, these data show that HSV-2 is common among pregnant women in Africa [22, 25]. In comparison to seroprevalence reported in middle and high income countries like India and Italy, the seroprevalence reported in the current study is indeed high [26, 27]. This might be attributable to the improved health care, awareness and healthy seeking behavior in developed countries compared to rural areas of Tanzania and other low income countries. Compared to previous studies in Dar es salaam and Moshi which focused on urban population the findings are comparable to the current study [28, 29]. However, the observed seroprevalence in the current study is slightly higher than what was reported in Manyara and Singida [21, 30]. The differences could be explained by different diagnostic methods used where by the previous studies used non-commercial peptide-55 Enzyme-linked immunosorbent assay.

Considering the fact that in Tanzania only HIV and syphilis are screened during antenatal visits, this calls for the need for policy makers to consider inclusion of HSV-2 screening in the Tanzanian antenatal package which overall will improve antenatal care in Tanzania. In this study about 5% of women were HSV-2 IgM seropositive indicating acute or recent infection which put unborn child at an increased risk of vertical transmission that can lead to neonatal herpes [5, 7]. This emphasizes the need of considering screening of HSV-2 during antenatal visits so that care can be taken in handling these women during delivery. Due to the nature of the current study these women were not followed up to establish outcomes of the newborns. Further studies to determine the outcomes of maternal HSV-2 infection are warranted in this setting. In addition, eight women who were HSV-2 IgG seropositive were found to have genital ulcer whereby 4 of them were *T. pallidum* (syphilis) seropositive. This indicates that other STIs are also common among pregnant women in rural areas of Mwanza.

Among the factors studied in this study, a history of sexually transmitted infection was found to predict IgM seropositivity which is similar to the previous reports [22, 28, 30]. In the current study no significant association was established between HIV and HSV-2 seropositivity which is contrary to what was observed in the previous studies [31, 32]. This could be explained by a small number of HIV seropositive women in the current study.

### Prevalence and associated factors of HBsAg among pregnant women in rural areas of Mwanza city

In this study the prevalence of HBsAg was found to be 5.8% which is almost three folds higher than a previous study in an urban areas of Moshi municipal which reported the prevalence of 2.0% and two folds higher than another study in urban areas of Mwanza city which reported the prevalence of 3.8% [33, 34]. Our results show that HBV is more prevalent in rural areas than urban areas of Tanzania. The possible explanation could be exposure to the risk factors which might be associated with knowledge gap of possible routes of HBV transmission. As reported previously [35–37], most of rural residents do not have enough knowledge on HBV transmission as compared to urban counterparts. In comparison to previous reports in African countries; Sierra Leone, Mali, Kenya, Nigeria and Burkina Faso which reported prevalence of 6.2, 8.0, 8.8, 11 and 17.2% respectively, the prevalence reported in the current study is low [38–42]. The possible geographical variations could play a role in these differences and exposure to the risk factors as well as different tests used in these studies. The current study used rapid immunochromatographic test with sensitivity and specificity of > 98% which might be different in other studies.

Regarding the factors associated with HBV infection, in the current study only parity was found to independently predict HBV positivity. Increase in age was found to be associated with HBV infections on univariable analysis, this observation is inconsistent to a previous study in Sudan which did not find any association of HBV infection with age among pregnant women [43]. Nevertheless, the findings in the current study are in line with other studies in Burkina Faso, Nigeria and Moshi municipal [34, 40, 41]. As previously reported prevalence of viral infections tends to increase with increase in age [44, 45] hence aged women who are also likely to have more parity are more likely to be exposed to the risk factors than their counterparts.

### Prevalence and associated factors of HCV among pregnant women in rural areas of Mwanza city

Regarding HCV seropositivity, the seroprevalence was found to be 0.04% which is in agreement with previous studies in Sudan and Nigeria [46, 47]. In comparison to previous reports in Egypt, Ivory coast and Rwanda which reported the prevalence of 6.1, 3.6 and 2.4% [46, 48], the reported HCV seroprevalence in this study was significantly low. This could be explained by different methods used in these studies which might significantly differ in sensitivity and specificity. In the current study, the test used has sensitivity and specificity of 98.9 and 99.2% which might be different from other previous studies. Another possible explanation could be variations in geographical factors which might influence endemicity status in different areas [43, 46, 48–50].

### Prevalence and associated factors of HIV seropositivity among pregnant women in rural areas of Mwanza city

In the current study, the prevalence of HIV was found to be 5.0% which is comparable to a previous study by Manyahi et al. that reported the prevalence of 5.6% among pregnant women in urban and rural settings of Tanzania [51]. This observation is also supported by another study by Malima et al. which reported a slight low HIV prevalence (2.6%) among pregnant women in rural setting in Northern part of Tanzania [52]. In comparison to HIV prevalence in general population (5.3%) in Tanzania mainland, the reported prevalence of HIV in the current study is comparable to the general population [19]. These findings indicate that the prevalence of HIV among pregnant women in Tanzania is almost similar between urban and rural settings as well as in general population which could be explained by ongoing interventions on HIV control across the country. In comparison to previous reports in Rwanda and Zambia which documented the prevalence of 25.4 and 24.1%, respectively [53, 54], the reported prevalence in the current study is indeed low. The difference could be explained by the fact that the previous studies were perhaps conducted before a wide spread HIV control intervention across the continent. Our findings are also in agreement with other studies in the Sub-Saharan African countries [51, 55, 56] which reported declining trends in HIV among pregnant women population in recent years [55, 57].

Among the factors tested, only positive *T. pallidum* serostatus predicted HIV seropositivity among pregnant women in rural settings of Mwanza region which is inconsistent with a previous study [51]. The possible explanation could be sensitivity and specificity of the test used to diagnose *T. pallidum* among these studies. In the current study rapid immunochromatographic test and TPHA were used with sensitivity and specificity of > 97% which could be different from a previous study. Nevertheless, the current study are in agreement with previous studies which reported a significant association between these two infections [58–61]. This could be explained by the fact that HIV and *T. pallidum* infections shares the same transmission route whereby occurrence of one infection is likely to be influenced by another infection.

In this study despite lower abdominal pain being asked was not included in the analysis of factors and in the definition of history of STIs because it is non-specific symptom of STIs**.** This might have underestimated the history of STIs magnitude. In addition, because the recruitment was done during antenatal visits the population did not represent community pregnant women because the ANC attendance is about 73%**.** Finally, the diagnosis of HSV based on rapid immunochromatographic tests which are not as sensitive as Enzyme Immuno-assays, therefore the prevalence might be underestimated.

## Conclusion and recommendations

In conclusion, a significant proportion of pregnant women in rural settings of Mwanza region is infected with at least one sexually transmitted viral infection which is predicted by positive *T. pallidum* serostatus. Seroprevalence of HSV-2 IgG antibodies among pregnant women residing in rural areas of Mwanza Tanzania is alarmingly high and is predicted by history of sexual transmitted infections. In addition, HSV-2 IgG seroprevalence in the current study was significantly high among women aged below 20 years compared to those aged above 20 years. Further studies in other areas of Tanzania and studies to establish the outcome of pregnancy in HSV-2 IgM seropositive women in rural population are highly recommended. Moreover, the prevalence of HBV infection among pregnant women in rural areas of Mwanza region is high while the prevalence of HCV is relatively low compared to other settings. These findings call for the need to strengthen the current antenatal screening protocol by including other viral infections that can potentially results into adverse pregnancy outcomes.

## Supplementary information

**Additional file 1.** Data collection tool.

## Data Availability

The datasets used and/or analysed during the current study are available from the Director of Research and Publication of the Catholic University of Health and Allied sciences on reasonable request.

## Abbreviations

CI: Confidence interval
HBsAg: Hepatitis B surface antigen
HBV: Hepatitis B virus
HCV: Hepatitis C virus
HIV: Human immunodeficiency virus
HSV-2: Herpes simplex virus − 2
IgG: Immunoglobulin gamma
LICs: Low income countries
OR: Odds ratio
