# Supplementary material for: Treponema pallidum infection predicts sexually transmitted viral infections (hepatitis B virus, herpes simplex virus-2, and human immunodeficiency virus) among pregnant women from rural areas of Mwanza region, Tanzania
Source: BMC Pregnancy Childbirth. 2019 Oct 29;19:392. doi: 10.1186/s12884-019-2567-1 (PMC6820934; doi:10.1186/s12884-019-2567-1)
Supplement: Supplementary file 1 — Additional file 1. Data collection tool. [file 12884_2019_2567_MOESM1_ESM.pdf]

DATA COLLECTION TOOL

QN	CODE	QUESTION AND FILTER	CODING CATEGORY
**PATIENT PARTICULARS**			
01	INITIALS	Write interviewer's initials	_____ _____
02	DATE	Write date of interview	--------/----------/---------<br>Day Month Year
03	STUDY NUMBER	Number	_______________
04	PHONE	Contact phone number(s)	Women: ________________________<br>Next of kin: _____________________
**SOCIODEMOGRAPHIC INFORMATION**			
05	AGE	What is your age?	___________________Years
06	RESIDENCY	Residence	___________________
07	RELIGION	Which religion are you believing?	i. Christian<br>ii. Muslim<br>iii. Others (specify)________________
08	MARITAL STATUS	What is your current marital status?	i. Married(monogamy)<br>ii. Married(polygamy)<br>iii. Cohabiting<br>iv. Widowed<br>v. Separated<br>vi. Single(never married)
09	EDUCATION LEVEL	What is the highest level of formal education you have completed?	i. Never attended school<br>ii. Complete primary school<br>iii. dropped out of school<br>iv. completed Secondary school<br>v. Still in school<br>vi. University/College
10	OCCUPATION	Which of the following best describes your main work status in the past 12months?	i. Government Employed<br>ii. Unemployment<br>iii. House wife<br>iv. Animal keeper<br>v. Peasants<br>vi. Petty trader<br>vii. Others (specify)______________
**OBSTETRICS HISTORY**			
11	PARITY	Number of child birth	_____
12	LNMP	What is the first day of your last normal menstrual cycle?	--------/-----------/---------<br>Day Month Year
13	EXPECTED DATE OF DELIVERY	What is the expected date of delivery?	--------/-----------/---------<br>Day Month Year
14	GESTATION AGE	What is the gestation by dates-in completed weeks?	--------------------------weeks

GYNAECOLOGICAL HISTORY AND OTHER RISK FACTORS			
15	SEXUAL HISTORY	Age at first sexual encounter	______________years
16	NUMBER OF SEXUAL PARTNERS	Number of sexual partners for the past 1 year?	________________
17	PARTNENER'S WOMAN OUTSIDE RELATIONSHIP	Partner has other women outside of the relationship?	i. YES<br>ii. NO
18	NUMBER OF YEARS IN RELATIONSHIP	Number of years by which sexual partner is older?	__________years
19	CONTRACEPTION USE	Have you ever use contraception?	i. YES<br>ii. NO
20	CONTRACEPTION TYPE	If yes above which type of contraception?	______________________________
21	H/CONDOM USE	Do you have history of use condom as your mode of contraception before conception?	i. YES<br>ii. NO
22	STI'S SYMPTOMS	Have you had any of the following complains recently or in the past?	i. Abnormal per vagina discharge<br>ii. Painful micturition<br>iii. Lower abdominal pain<br>iv. Painful coitus<br>v. Genital ulcers or blisters<br>vi. No symptoms
23	PREVIOUS STI'S HISTORY	Previous history of STI's?	i. YES<br>ii. NO
24	CLINICAL GENITAL ULCER	Visual inspection of genital ulcer or blister	i. YES<br>ii. NO
25	LOW BIRTH WEIGHT	History of baby with low birth weight?	i. YES<br>ii. NO
26	STILLBIRTH	History of stillbirth?	i. YES<br>ii. NO
27	MISCARRIAGES	History of miscarriages in pervious pregnancies?	i. YES<br>ii. NO
28	TATTOOS/TRADITIONAL MARKS	Presence of tattoos/traditional marks?	i. YES<br>ii. NO
29	BLOOD TRANSFUSION	History of blood transfusion?	i. YES<br>ii. NO
30	INTRAVENOUS DRUG USE	History of intravenous drug use?	i. YES<br>ii. NO
31	NEEDLE STICK INJURIES	History of needle sticks injuries?	i. YES<br>ii. NO
32	SURGERY	History of surgery?	i. YES<br>ii. NO
33	ORGAN TRANSPLANT	History of organ transplant?	i. YES<br>ii. NO
34	SHARING SHARP OBJECTS	History of sharing sharp objects?	i. YES<br>ii. NO
35	NASAL DRUG EQUIPMENT	History of sharing nasal drug consumption equipments?	i. YES<br>ii. NO

36	TEETH EXTRACTION	History of teeth extraction?	i. YES<br>ii. NO
37	HEALTH STATUS	PMTCT status?	i. One<br>ii. Two
38	SYPHILIS TEST	Laboratory results of *Treponema pallidum* (syphilis)	Non-Treponemal antibody test<br>i. Reactive<br>ii. Non reactive<br>Treponemal test<br>i. Reactive<br>ii. Non reactive
39	HSV-2 RAPID TEST	Laboratory results of HSV-2 rapid test	IgG<br>i. Reactive<br>ii. Non reactive<br>IgM<br>i. Reactive<br>ii. Non reactive
40	HBV	Laboratory results of HBsAg	i. Reactive<br>ii. Non reactive
41	HCV	Laboratory results of HCV rapid test	i. Reactive<br>ii. Non reactive
42	HIV	Laboratory results of HIV rapid test	i. Reactive<br>ii. Non reactive
